# The influence of fasting and caloric restriction on inflammation levels in humans: A protocol for systematic review and meta analysis

**DOI:** 10.1097/MD.0000000000025509

**Published:** 2021-04-16

**Authors:** Rui-han Zhou, Qian Wang, Xiao-min Hu, Mei Liu, An-Ren Zhang

**Affiliations:** a School of Health Preservation and Rehabilitation, Chengdu University of Traditional Chinese Medicine; b Care Alliance Rehabilitation Hospital of Chengdu; c Department of Rehabilitation Medicine, Shanghai Fourth People's Hospital Affiliated to Tongji University School of Medicine, China.

**Keywords:** caloric restriction, fasting, humans, inflammation, meta-analysis, protocol, systematic review

## Abstract

**Background:**

Fasting and caloric restriction have a potential means of anti-inflammatory, as they can decrease the level of systemic inflammation. Although encouraging results have been obtained in animal experiments, there is no consensus on whether these results are applicable to human. The objective of this systematic review and meta-analysis is to analyze the influence of fasting and caloric restriction on inflammation levels in humans.

**Methods:**

The systematic review and meta-analysis will be conducted in accordance with the Preferred Reporting Items for Systematic Review and Meta-Analysis (PRISMA) guidelines. The following eight databases will be searched:(The retrieval time is from the establishment of each database to December 2020): PubMed, the Cochrane Library, Embase, Web of Science, China National Knowledge infrastructure (CNKI), China Biology Medicine (CBM), Wan Fang Data, the Chinese Science and Technology Periodical Database (VIP). Relevant data will be performed by Revman 5.3 software provided (Cochrane Collaboration) and Stata 14.0 statistical software.

**Results:**

The results of this systematic review and meta-analysis will be published in a peer-reviewed journal.

**Conclusions:**

This systematic review will provide evidence to judge the effectiveness of fasting and calorie restriction in human subjects, so as to provide a sound basis for future research and lifestyle promotion.

**INPLASY registration number:**

INPLASY202130026

## 1 Introduction

The increase in life expectancy worldwide has not paralleled the same increase in healthy aging. Developed and developing countries are facing social and economic challenges brought about by the disproportionate growth of the elderly population and the accompanying burden of chronic diseases.^[1]^ Dietary restriction therapies have emerged as powerful tools to ameliorate and postpone the onset of disease and delay aging.^[2]^ According to meal size and frequency, dietary restriction therapies are divided into two categories:

1. Classical caloric restriction, in which daily caloric intake is typically decreased;
2. Fasting, intermittent or periodic full or partial fasting, that is a periodic, full-or multiday decrease in food intake, usually accompanied by a decrease in energy intake; or fasting without decreasing in energy intake, enough energy is taken in within the limited time window, thereby prolonging the fasting time.

As other researchers reported,regardless of whether energy intake is reduced or not, as long as the fasting time is prolonged, it can have profound health benefits.^[3]^

Inflammation is a common biological immune response to pathogenic factors in immune system. It is triggered by stimulus of viral, fungal, and bacterial origins. Chronic inflammation is an unresolved, low-grade inflammatory response of the innate immune system. It may result in constant inflammatory response and abnormal innate immune response. Accumulating evidence supports that low-grade systemic chronic inflammation plays an important role in regulating age-related disease such as dementia, obesity, type 2 diabetes, atherosclerosis, osteoporosis, sarcopenia, and others.^[4]^ Fasting and caloric restriction decrease inflammation, which are believed to protect against age associated diseases.^[5]^ It was found in animal models that fasting and caloric restriction can markedly alleviate systemic inflammation due to its anti-inflammatory properties in various tissues. For instance, in obese mice, caloric restriction significantly decreased mRNA expression levels of inflammatory cytokines and chemokines in white adipose tissue, including IL-6, IL-1Ra, IL-2, MCP-1, and CXCL16.^[6]^ In mice liver, even mild CR reduces inflammatory gene expression.^[7]^

Although encouraging results have been obtained in animal experiments, there is no consensus on whether these results are applicable to human. The meta-analysis of evidence-based medicine by systematically reviewing the existing literature is to analyze the influence of fasting and caloric restriction on inflammation levels in humans. In order to further evaluate the efficacy of fasting and calorie restriction in promoting health in humans and provide reference for follow-up research.

## 2 Methods and analysis

### 2.1 Study registration

The protocol has been registered on the INPLASY register. The registration number is INPLASY202130026 (Available from: https://inplasy.com/inplasy-2021-3-0026/). This protocol of meta-analysis will be reported in accordance with the preferred reporting items of the system review and meta analysis (PRISMA) statement guidelines.^[8,9]^

### 2.2 Eligibility criteria

The participant, intervention, comparison, outcome, time, and study design (PICOTS) criteria was used to establish study inclusion criteria.

#### 2.2.1 Types of study

We will include all randomized controlled trials of fasting or caloric restricted diets in human subjects. No restrictions on language.

#### 2.2.2 Type of participants

All included cases must be healthy adults or diseased patients (over 18 years old). No restrictions about sex and ethnicity was applied in the study population.

#### 2.2.3 Type of interventions

Fasting or caloric restriction diets will be used as interventions.

1. Classical caloric restriction, in which daily caloric intake is typically decreased;
2. Fasting, intermittent or periodic full or partial fasting, that is a periodic, full-or multiday decrease in food intake, usually accompanied by a decrease in energy intake; or fasting without decreasing in energy intake, enough energy is taken in within the limited time window, thereby prolonging the fasting time.

#### 2.2.4 Type of comparators

In the same original study, the subjects on a normal diet, and the rest of the health conditions were the same as those of the experimental group.

#### 2.2.5 Types of outcome measurements

Inflammatory factors in the circulating blood will be the primary outcome reflecting the level of inflammation in humans. The main markers of inflammation include:IL-1β, IL-6, TNF-α, high-sensitivity C-reactive protein (hs-CRP) and so on. Secondary results includes the total WBC and lymphocyte counts, ICAM-1, leptin, etc.

### 2.3 Exclusion selection

Any one of the following articles can be excluded:

1. Animal experiments or cell experiments, not human experiments;
2. Observational studies, animal experiments, case reports, reviews and other non-randomized controlled trials or self-control study or random method error research investigation;
3. For the repetitive articles, only those with the latest publication year, large sample size and comprehensive information are retained;
4. Unable to extract valid outcome data;
5. Studies that only changed the composition of the diet without reducing energy intake or extending fasting time.

### 2.4 Search strategy

We will search the following databases (The retrieval time is from the establishment of each database to December 2020): PubMed, the Cochrane Library, Embase, Web of Science, China Biology Medicine (CBM), China National Knowledge infrastructure (CNKI), Wan Fang Data, the Chinese Science and Technology Periodical Database (VIP). The group of search words was a combination of human, inflammation and caloric restriction (or fasting or restriction caloric or low-calorie diet or intermittent fasting or hunger strike or time restricted feedings or fasting intermittent). Take PubMed as an example to list the retrieval strategy, which will be attached as Annex 1(https://www.kdocs.cn/l/so0GXtgkxWxD).

### 2.5 Studies selection

**Figure 1:**
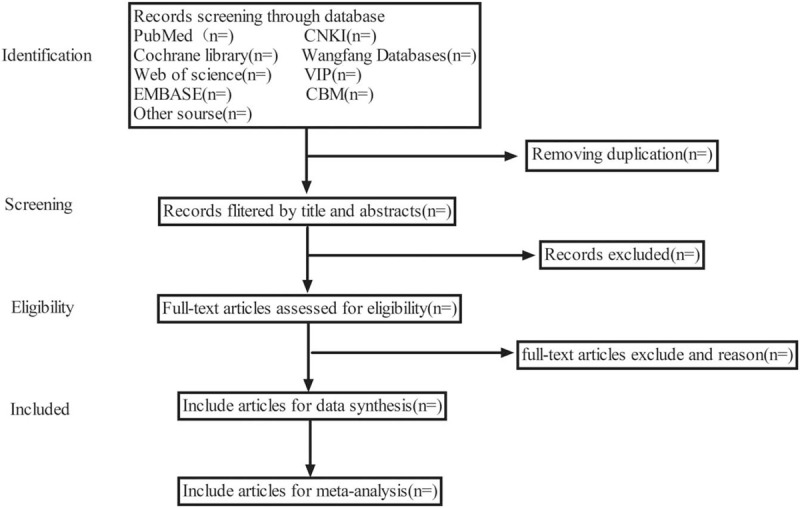
Flow diagram of studies identified.

EndNote X9 was used for citation management. Duplicates were excluded using the Endnote function “remove duplicates”. Two independent authors investigated the abstract of all articles to exclude the obviously irrelevant literature, animal experiments, reviews, case reports, etc. After the first step of screening, the two researchers obtained the full text for more detailed reading inorder to determine whether they meet the inclusion criteria. After that, the two researchers cross checked the results of the included study. The divergent studies will be discussed by two researchers or decided by a third researcher. The procedures of study selection was performed in accordance with the PRISMA flowchart (see Fig. 1).

### 2.6 Quality assessment

Two researchers selected the quality assessment methods provided by Cochrane Handbook, including risk bias assessment form and Jadad modified scale. In case of disagreement, it will be decided by a third researcher. According to the methodology of randomized controlled trial, the following evaluation items will be adopted: random sequence generation, allocation concealment, blind methods, results data integrity, selective outcome reporting and other bias. As a result, the quality of evidence will be accepted as low risk, high risk, or ambiguous bias risk.

### 2.7 Data extraction

Two reviewers are responsible for information extraction according to the following information: The basic information of the included studies, including the first author, the year of publication, etc; The basic characteristics of the subjects, including the number of subjects in the experimental group and the control group, gender composition, average age; Type of intervention, fasting or caloric restriction, percent of caloric restriction, treatment course and other specific details; Outcome measurement data needed for the study.

### 2.8 Data analysis

Relevant data will be performed by Revman 5.3 software provided by the Cochrane Collaboration and Stata 14.0 statistical software. Relative risk (RR) will be used for dichotomy results with 95% confidence intervals, and Mean difference (MD) or normalized mean difference (SMD) will be used for continuous variables with 95% confidence intervals.

#### 2.8.1 Assessment of heterogeneity

The choice of random effect model or fixed effect model depends on the heterogeneity of the original research. In this study, the Cochrane Q test and I^2^ will be used to analyze the heterogeneity between studies, If there is no heterogeneity (I^2^<50%, *P* > .1), fixed effect model will be used in meta analysis. Otherwise, we will choose subgroup analysis, sensitivity analysis or meta regression to explore the causes of heterogeneity. If the cause cannot be found and the degree of heterogeneity is acceptable, the random effect will be selected.

##### 2.8.1.1 Subgroup analysis

Subgroup analysis was performed to identify the source of heterogeneity among trials. In this study, type of intervention (fasting or caloric restriction), percent of caloric restriction (less than 50% or more than 50% daily requirement caloric intake) and the health of the subject (healthy subjects or patients with disease) were considered as predefined source of heterogeneity.

##### 2.8.1.2 Sensitivity analysis

Sensitivity analysis is to explore the impact of individual studies on aggregate results, which will be judged by the method of excluding studies one by one, to check the robustness of the comprehensive results.

#### 2.8.2 Publication bias

Funnel plot, Begg tests, Egger test will be used to investigate whether there is publication bias in this study. If the funnel plot is asymmetric, we will continue to process the asymmetric funnel plot through the shear compensation method, to ensure the symmetry of the funnel plot and eliminate publication bias.

#### 2.8.3 Quality of evidence

Grading of Recommendations Assessment, Development, and Evaluation (GRADE) system will be used to evaluate the quality of evidence for the outcomes. Comprehensive results caused by five factors (risk of bias, inconsistency, indirectness, imprecision, publication bias) affect the quality of evidence, which is divided into four levels: high level, medium level, low level, very low level.^[10,11]^

### 2.9 Ethics and dissemination

No ethical approval was required, as SR will be based on published research. According to the PRISMA guidelines, SR's results will be published in a peer-reviewed scientific journal.

## 3 Discussion

Aging is a complex and progressive process characterized by physiological and functional decline with time that increases susceptibility to diseases. Aged-related functional change is accompanied by a low-grade, unresolved chronic inflammation as a major underlying mechanism. As a healthy and economical lifestyle, both fasting and caloric restriction diets are worthy of attention. Fasting and calorie restriction have shown definite anti-aging effects in rodents^[12]^ and non-human primates.^[13,14]^ In addition, in animal models, fasting and calorie restriction decrease inflammation, which is believed to protect against age-related diseases. Given these promising results found in animal experimental models, the transferability of such findings into humans is of paramount interest.

This article will be the first review on the systematic evaluation of the influence of fasting and caloric restriction on inflammation levels in human subjects. It has drawn reasonable conclusion by collecting evidence, sorting out and analyzing data about the effectiveness of fasting and calorie restriction in human subjects, so as to provide a sound basis for future research and lifestyle promotion.

## Acknowledgments

We would like to thank the members who have devoted themselves to this research.

## Author contributions

**Conceptualization:** An-Ren Zhang.

**Methodology:** Rui-han Zhou, Qian Wang.

**Supervision:** Xiao-min Hu, Mei Liu.

**Writing – original draft:** Rui-han Zhou.
